# Learning process for identifying different types of communication via repetitive stimulation: feasibility study in a cultured neuronal network

**DOI:** 10.3934/Neuroscience.2019.4.240

**Published:** 2019-10-16

**Authors:** Yoshi Nishitani, Chie Hosokawa, Yuko Mizuno-Matsumoto, Tomomitsu Miyoshi, Shinichi Tamura

**Affiliations:** 1 Department of Radiology, Graduate School of Medicine, Osaka University, Suita 565-0871, Japan; 2 Graduate School of Science Osaka City University, Osaka, 558-8585, Japan; 3 Graduate School of Applied Informatics, University of Hyogo, Kobe 650-0044, Japan; 4 Department of Integrative Physiology, Graduate School of Medicine, Osaka University, Suita 565-0871, Japan; 5 NBL Technovator Co., Ltd., 631 Shindachimakino, Sennan 590-0522, Japan

**Keywords:** cultured neuronal network, spike wave propagation, identifying communications, repeated stimulations, learning process

## Abstract

It is well known that various types of information can be learned and memorized via repetitive training. In brain information science, it is very important to determine how neuronal networks comprising neurons with fluctuating characteristics reliably learn and memorize information. The aim of this study is to investigate the learning process in cultured neuronal networks and to address the question described above. Previously, we reported that the spikes resulting from stimulation at a specific neuron propagate as a cluster of excitation waves called spike wave propagation in cultured neuronal networks. We also reported that these waves have an individual spatiotemporal pattern that varies according to the type of neuron that is stimulated. Therefore, different spike wave propagations can be identified via pattern analysis of spike trains at particular neurons. Here, we assessed repetitive stimulation using intervals of 0.5 and 1.5 ms. Subsequently, we analyzed the relationship between the repetition of the stimulation and the identification of the different spike wave propagations. We showed that the various spike wave propagations were identified more precisely after stimulation was repeated several times using an interval of 1.5 ms. These results suggest the existence of a learning process in neuronal networks that occurs via repetitive training using a suitable interval.

## 1. Introduction

It is well known that various types of information can be learned and memorized. These processes are based on the functions of large neuronal networks that are assembled through spike propagation (action potentials) via synapses [1]–[5] in the brain. These functions are very complex and ambiguous. In such conditions, how can multiple types of information be communicated and stored? How do neuronal networks function in the learning process? These are very important, yet difficult, topics for brain function. Many researchers have attempted to answer these questions. Most previous studies were based on spike-coding metrics [6], spatiotemporal coding models [7]–[13], and synchronous action models [14]–[18]. Regarding data communication in the brain, we reported that spikes propagating from stimulated neurons are received by afferent neurons as random-like sequences in simulated and natural asynchronous neuronal networks [19]–[25]. This phenomenon is similar to radio wave propagation in artificial data communication systems; hence, this phenomenon was termed “spike wave propagation.” In those studies, we showed that stimulated neurons were able to identify various spatiotemporal patterns of spike wave propagation in specific areas (receiving area) of the neuronal network. These results indicated that different types of communication can be identified. From a basic brain function point of view, we consider that the identification of types of communication is related to the identification of different objects using vision by seeing, of different sounds using hearing, etc. [26]–[27]. In contrast, from a learning function point of view, we can expect that the quality of the identification of the different stimulation areas is improved by repeating the stimulation corresponding to the learning process, as they can be identified more accurately via repeated training. However, this has not been confirmed because our previous experiments [24]–[25] used single stimulation exclusively.

Here, we investigated this learning process using repetitive stimulation in cultured neuronal networks as a feasibility study of learning function. This work is an application of the results of our recent study [24]–[25].

## 2. Materials and methods

### 2.1. Cell culture recording

Cell culture, stimulated spike recording, and coding spike trains were as described previously [24]–[25]. Hippocampal neurons were dissected from Wistar rats on embryonic day 18. The procedure conformed to the protocols approved by the Institutional Animal Care and Use Committee of the National Institute of Advanced Industrial Science and Technology. Cells were cultured individually on an array of 64 planar microelectrodes (8 × 8 configuration). Each electrode had an individual number, from channel 1 (termed ch1 henceforth) to ch64, and corresponded to one neuron as shown in Figure S1 in Supplementary Information. In this study, we prepared 11 cultures, which were termed culture 01–11.

### 2.2. Stimulation and coding spike trains

The stimulation method used here was as follows.

**Figure 1.**
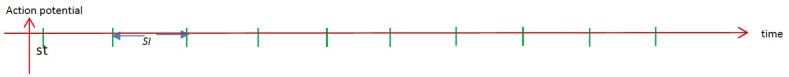
Repetition of the stimulation using a constant stimulation interval (*SI*). Stimulation (st; indicated by 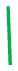)

(1) Stimulation was repeated 8–12 times at a particular stimulation interval (*SI*) at one electrode (corresponding to one neuron), as shown in Figure 1.

(2) The procedure described in (1) was performed at an additional two electrodes.

(3) Procedures (1) and (2) were repeated 5–10 times for the back-propagation neural network (BPNN) analysis (see Section 2.3).

In this study, we set SI to 0.5 and 1.5 s and compared the results obtained. The stimulation channels used (termed St*ch* henceforth) were ch11, 36, and 54.

**Figure 2.**
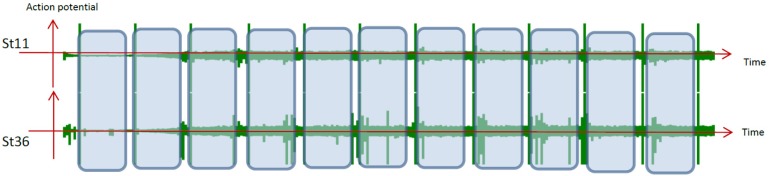
Investigation of the timing of the stimulation for the confirmation of *Identifiable*. 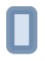
 is the analysis domain for *Identifiable* where a spike train caused by stimulation is observed. *Identifiable* are neurons that can identify spike patterns stimulated by different channels (St*ch*). For example, this figure shows st11 vs. st36. *Identifiable* channels are checked after each stimulation.

As described in [25], the time sequence data used for identifying a stimulated neuron (channel) were coded as stimulated spike trains at specific neurons (current channels) and their adjacent (to the left and right) neurons (see Figures 2 and 3(b) in [25]).

### 2.3. Investigation of the effect of repeated stimulation on classification

We analyzed the temporal patterns of the spikes generated by each stimulation (Figure 2). Subsequently, we investigated the channels that could be used to identify a spike pattern that was stimulated by two different St*ch* (see Section 2.2). Henceforth, we will refer to such a channel as *Identifiable*. The classification method was the BPNN machine learning method, similar to that described in [25].

**Figure 3.**
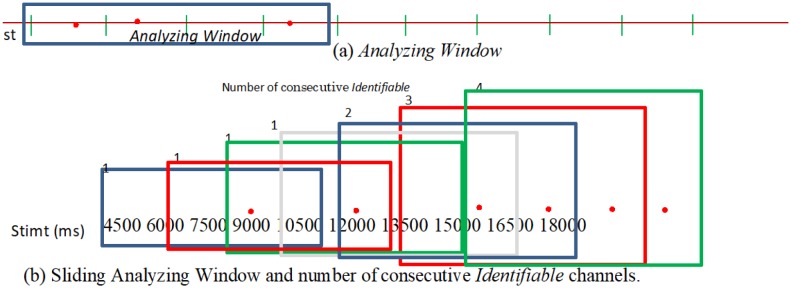
Analytical method used to determine the variation in the number of consecutive *Identifiable* channels. Color 
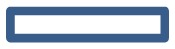
 indicate analyzing window. 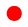 indicates *Identifiable*.

If a learning process occurs in the neuronal network, the number of *Identifiable* channels that can identify a spike pattern stimulated by different channels must be increased by repeated stimulation. Therefore, we analyzed the variation in the number of continuous *Identifiable* channels after repeated stimulation in each channel, as follows. First, we set an *Analyzing Window* including four stimulations in a time series (see Figure 3(a)) and counted the number of consecutive *Identifiable* channels in this window. For example, the number of consecutive *Identifiable* channels was 2 in Figure 3(a). Next, these procedures were repeated by sliding the *Analyzing Window* in the direction of the temporal axis (by stimulation). The outline of this procedure is shown in Figure 3(b).

## 3. Results and discussion

**Figure 4.**
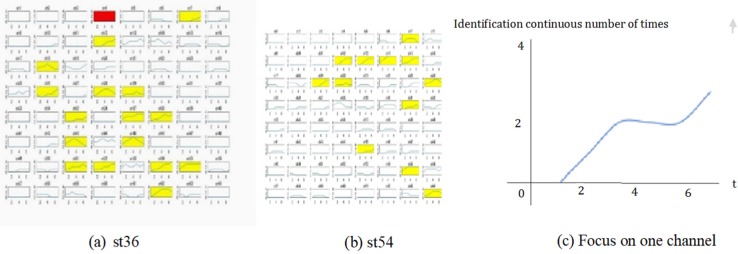
Time variation of *Identifiable*, st36 vs. st54, in each channel in culture 07 (SI was 1.5 s). The array of channels in (a) and (b) corresponds to the deposition of electrodes in MED-P545A (see Figure S1 in Supplementary Information). The horizonal axis represents the sliding of the analyzing window in the direction of time. The scale of this axis corresponds to the repeated number of stimulations. The vertical axis is the number of *Identifiable* channels. The cells in yellow are channels for which the number of *Identifiable* channels was increased by 2 or more after repeated stimulations. The cells in red are channels for which the number of SI was decreased by 2 or more after repeating stimulations (contrary to our assumption).

Figure 4 shows the variation of consecutive *Identifiable* channels with time (see Section 2.3) in each channel in culture 07 when st*ch* was ch36 and ch54 and *SI* was 1.5 s. The horizonal axis represents the position of the *analyzing window*, which corresponds to the timing of the stimulation. The vertical axis represents the number of consecutive *Identifiable* channels. The results depicted in this figure showed that the number of consecutive *Identifiable* channels increased in some channels.

To investigate the spatial distribution of such channels, we colored cells in yellow and red (the meaning of these colors is described in the caption of this figure). Channels for which an increment in the number of consecutive *Identifiable* channels was observed (termed *yellow cells* henceforth) gathered in a particular area, which was comparatively wide. In contrast, a decrement in the number of consecutive *Identifiable* channels (termed *red cells* henceforth) was also observed in some channels; however, the number of *red cells* was much smaller than that of *yellow cells*.

**Figure 5.**
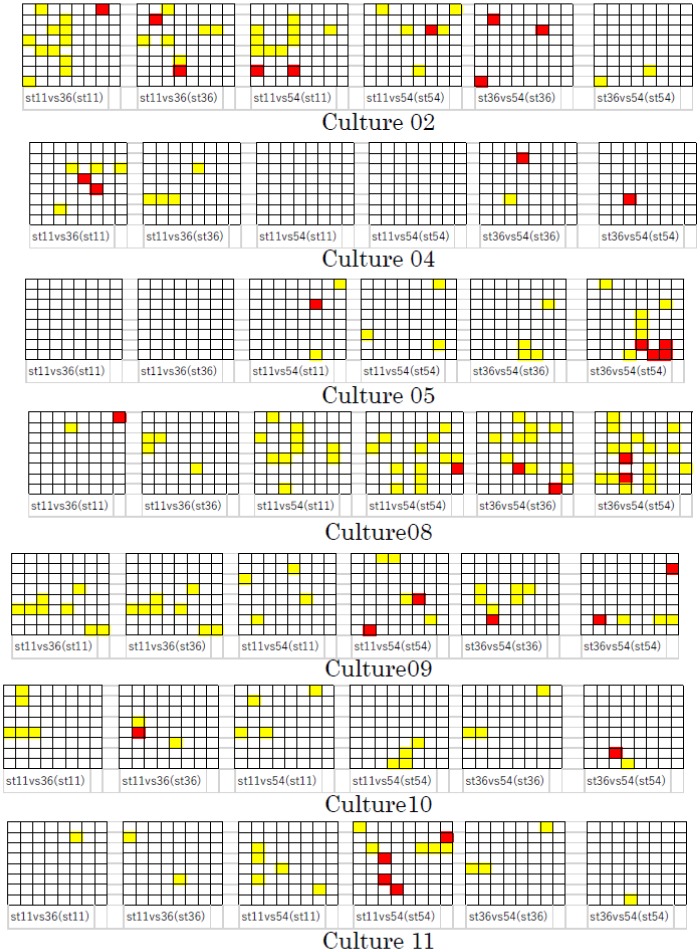
Distribution of *yellow cells* and *red cells* in other cultures (*SI* was 1.5 s). The meaning of *yellow cell* and *red cell* is as in Figure 4.

We also applied this process to other cultures and all combinations of St*ch* (St11 vs. St36, St36 vs. St54, and St11 vs. St54) at an *SI* of 0.5 or 1.5 s. Figure 5 shows the distribution of *yellow cells* and *red cell* in these cultures when *SI* was 1.5 s. Because of space constraints, the time variation of consecutive *Identifiable* channels, as shown in Figure 4, was omitted. Although similar results were observed among these cultures, there was variation in culture and stimulation channel. Incidentally, the results obtained for cultures 01, 03, and 06 were not shown, as neither *yellow cells* nor *red cells* were observed in these cultures. We considered that these results depended on the individual properties of each cell, including the distribution of synaptic weight, refractory period, etc.

**Figure 6.**
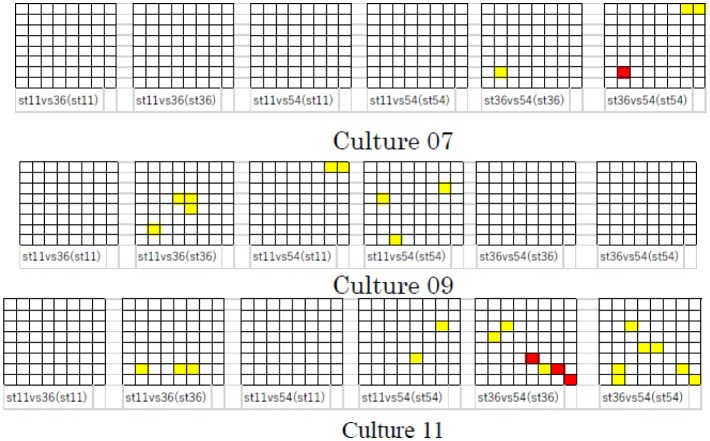
Distribution of *yellow cells* and *red cells* (*SI* was 0.5 s).

Figure 6 shows the distribution of *yellow cells* and *red cells* in these cultures when *SI* was 0.5 s. For this *SI*, *yellow cells* and *red cells* were observed only in cultures 07, 09, and 11. Moreover, the number of these cells was smaller than that observed for the *SI* of 1.5 s, even in these cultures (compare Figure 6 with Figure 5).

**Figure 7.**
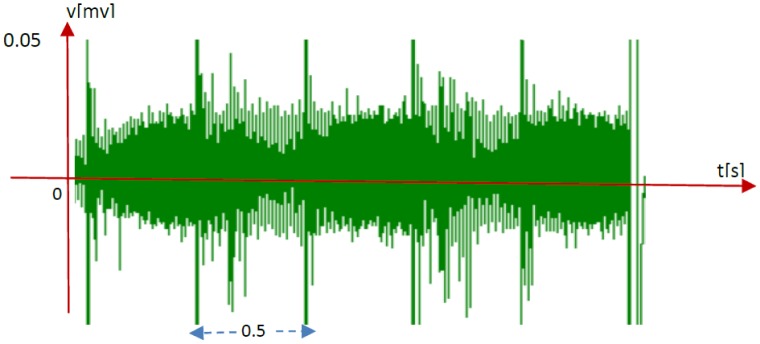
Overlap between a previous stimulation spike and new spikes. Extracted from row spike data (ch52, culture 07, 2^nd^ recording).

As an explanation for these results, it is possible that, for short stimulation periods, a few spikes resulting by the previous stimulation remain and overlap with the new spike, thus interfering with each other (see Figure 7).

Although repeated stimulation led to an increase in the number of *yellow cells*, it is unlikely that these phenomena happened accidentally. To address this issue, we performed the same analysis for imitation spike trains generated by random numbers. Few *yellow cells* and *red cells* were detected, which demonstrated that the results obtained for the cultures were not accidental.

Some learning processes are aimed at improving the identification of communications in a neuronal network via repeated stimulation in a particular area of the network. These learning processes are affected by the individuality of each cell. Although the reason for the existence of *red cells* (against our assumption) has not been clarified, it is possible that they are inhibitory neurons. Moreover, the effect of *SI* on the learning process is not clear, as we did not perform experiments using an *SI* longer than 1.5 s; however, it seems that the effect of the learning process was weakened, as the condition of the neuronal networks (distribution of synaptic weight, etc.) were reset before the stimulations.

We consider that these learning processes are similar to a repeated training aimed at learning information, to the skills of exercise, to learning how to play musical instruments, etc.

## 4. Conclusion

Our recent studies showed that stimulated channels were able to identify various spatiotemporal patterns of spike wave propagation in specific areas of the neuronal network. Here, we report that repeated stimulation increased the number of consecutive *Identifiable* channels at an *SI* of 1.5 s in a cultured neuronal network. This suggests that part of the learning process (learning knowledge or skill in view of basic brain activity) occurs via repetition training (stimulation). In contrast, this effect was barely observed for an *SI* of 0.5 s, because a few of the spikes elicited by the previous stimulation remain and overlap with the new spike, thus interfering with each other. As described above, spike repetition at an interval of 1.5 s yielded a superior result compared with an interval of 0.5 s for the identification of consecutive *Identifiable* channels. However, the effect of an *SI* longer than 1.5 s on the learning process has not been assessed. In future studies, we will examine the relationship between *SI* and learning process more clearly and identify the *SI* that is most suitable for the learning process. Moreover, we will investigate the learning process from the perspective of brain activity in greater detail.
