# Supplementary material for: Learning process for identifying different types of communication via repetitive stimulation: feasibility study in a cultured neuronal network
Source: AIMS Neurosci. 2019 Oct 16;6(4):240–9. doi: 10.3934/Neuroscience.2019.4.240 (PMC7179351; doi:10.3934/Neuroscience.2019.4.240)
Supplement: Supplementary file 1 [file neurosci-06-04-240-s001.pdf]

AIMS Neuroscience

AIMS Neuroscience, 6(4): 240–249.
DOI: 10.3934/Neuroscience.2019.4.240
Received: 04 July 2019
Accepted: 23 September 2019
Published: 16 October 2019

http://www.aimspress.com/journal/neuroscience

Research article

Learning process for identifying different types of communication via repetitive stimulation: feasibility study in a cultured neuronal network

Yoshi Nishitani[1,*], Chie Hosokawa[2], Yuko Mizuno-Matsumoto[3], Tomomitsu Miyoshi[4] and Shinichi Tamura[5]

[1] Department of Radiology, Graduate School of Medicine, Osaka University, Suita 565-0871, Japan
[2] Graduate School of Science Osaka City University, Osaka, 558-8585, Japan
[3] Graduate School of Applied Informatics, University of Hyogo, Kobe 650-0044, Japan
[4] Department of Integrative Physiology, Graduate School of Medicine, Osaka University, Suita 565-0871, Japan
[5] NBL Technovator Co., Ltd., 631 Shindachimakino, Sennan 590-0522, Japan

* **Correspondence:** E-mail: ynishitani1027@gmail.com; Tel: +819036276879.

Supplementary Figure

Figure S1 shows the deposition of electrodes in the microelectrode array, MED-P545A (Alpha MED Scientific, Kadoma, Osaka, Japan). The size of, and spacing between the electrodes was 50 × 50 μm^2 and 450 μm, respectively. Each electrode corresponds to a channel of spike recording. The number indicates the electrode (channel) number.

1	2	3	4	5	6	7	8
9	10	11	12	13	14	15	16
17	18	19	20	21	22	23	24
25	26	27	28	29	30	31	32
33	34	35	36	37	38	39	40
41	42	43	44	45	46	47	48
49	50	51	52	53	54	55	56
57	58	59	60	61	62	63	64

Figure S1. Deposition of electrodes in an MED-P545A microelectrode array.

AIMS Press

© 2019 the Author(s), licensee AIMS Press. This is an open access article distributed under the terms of the Creative Commons Attribution License (http://creativecommons.org/licenses/by/4.0)
